# Bcheck: a wrapper tool for detecting RNase P RNA genes

**DOI:** 10.1186/1471-2164-11-432

**Published:** 2010-07-13

**Authors:** Dilmurat Yusuf, Manja Marz, Peter F Stadler, Ivo L Hofacker

**Affiliations:** 1 Institute for Theoretical Chemistry, University of Vienna, Währingerstraße 17, A-1090 Wien, Austria; 2 Institut für Pharmazeutische Chemie, Philipps Universität Marburg, Marbacher Weg 6, D-35032 Marburg, Germany; 3 Bioinformatics Group, Department of Computer Science University of Leipzig, Härtelstrasse 16-18, D-01407, Leipzig, Germany; 4 Max Planck Institute for Mathematics in the Sciences, Inselstraße 22 D-04103 Leipzig, Germany; 5 Fraunhofer Institute for Cell Therapy and Immunology, Perlickstraße 1, D-04103 Leipzig, Germany; 6 Santa Fe Institute, 1399 Hyde Park Rd, Santa Fe, NM 87501, USA

## Abstract

**Background:**

Effective bioinformatics solutions are needed to tackle challenges posed by industrial-scale genome annotation. We present Bcheck, a wrapper tool which predicts RNase P RNA genes by combining the speed of pattern matching and sensitivity of covariance models. The core of Bcheck is a library of subfamily specific descriptor models and covariance models.

**Results:**

Scanning all microbial genomes in GenBank identifies RNase P RNA genes in 98% of 1024 microbial chromosomal sequences within just 4 hours on single CPU. Comparing to existing annotations found in 387 of the GenBank files, Bcheck predictions have more intact structure and are automatically classified by subfamily membership. For eukaryotic chromosomes Bcheck could identify the known RNase P RNA genes in 84 out of 85 metazoan genomes and 19 out of 21 fungi genomes. Bcheck predicted 37 novel eukaryotic RNase P RNA genes, 32 of which are from fungi. Gene duplication events are observed in at least 20 metazoan organisms. Scanning of meta-genomic data from the Global Ocean Sampling Expedition, comprising over 10 million sample sequences (18 Gigabases), predicted 2909 unique genes, 98% of which fall into ancestral bacteria A type of RNase P RNA and 66% of which have no close homolog to known prokaryotic RNase P RNA.

**Conclusions:**

The combination of efficient filtering by means of a descriptor-based search and subsequent construction of a high-quality gene model by means of a covariance model provides an efficient method for the detection of RNase P RNA genes in large-scale sequencing data.

Bcheck is implemented as webserver and can also be downloaded for local use from http://rna.tbi.univie.ac.at/bcheck

## 1 Background

In recent years, biological sequence databases have grown exponentially. These data include a rapidly increasing number of completely sequenced genomes as well as large-scale metagenomic data sets that await annotation. For instance, the Global Ocean Sampling Expedition (GOS) deposited more than 18G metagenomic sequences already [1]. The analysis of these data calls for new and more efficient methods of data analysis [2].

Non-protein-coding RNA (ncRNA) genes are abundant in genomic sequences, playing diverse important biological roles [3]. The genomic annotation of ncRNA genes is attracting strong research focus, in particular in the context of genome annotation [4,5] and metagenomics [6,7]. Methods for homology-based annotation have dramatically improved over the last years. In particular, Infernal 1.0 [8] outperforms previous methods by orders of magnitude in speed. Nevertheless, such general purpose approaches do not reach the performance levels of customized class-specific tools, in particular tRNAscan-SE[9] in terms of both speed and quality. Manual strategies in some cases [10] reach superior results, but are too time-consuming for larger projects and in most cases are hard to generalize.

tRNAscan-SE is not a single algorithm but rather a wrapper tool that combines a series of increasingly complex and expensive filters. Similarly, the major searching strategy of Rfam [11] is a combination of a blast-based filter followed by Infernal. The pre-filtering at sequence level with blast, however, is not ideal in particular in applications to distant homologs [12]. Another common approach is to apply a descriptor of sequence and structural motif to predict ncRNA homologs. The descriptor construction is a manual process requiring expert knowledge. Several descriptor languages have been developed, e.g. RNAmot [13], PatScan[14], HyPaL[15], RNAMotif[16], and rnabob[17], which is also used here.

RNase P RNA, possibly a remnant of the RNA world [18], is an important ribozyme involved in the processing of pre-tRNAs [19]. Its gene is usually designated as *rnpB *in bacteria. A variable number of protein components [20] facilitate substrate binding [21]. RNase P RNA exists in almost all organisms, but is absent in human mitochondrions [22]. So far, there is compelling evidence for the loss of RNase P RNA only in a single organism, the archaeon *Nanoarchaeum equitans *[18]. It is not unlikely, however, that plants, red algae, and heterokonts [23], some bacteria (e.g. *Aquifex aeolicus *[24,25]) and some additional archaea (e.g. *Pyrobaculum aerophilum *[24]) have lost their RNase P RNA. The archaeon *Methanothermobacter thermoautotrophicus *may be a transition towards the loss of RNase P RNA, which is catalytically inactive in this organism but can be "repaired" by a few substitutions [26].

**Figure 1:**
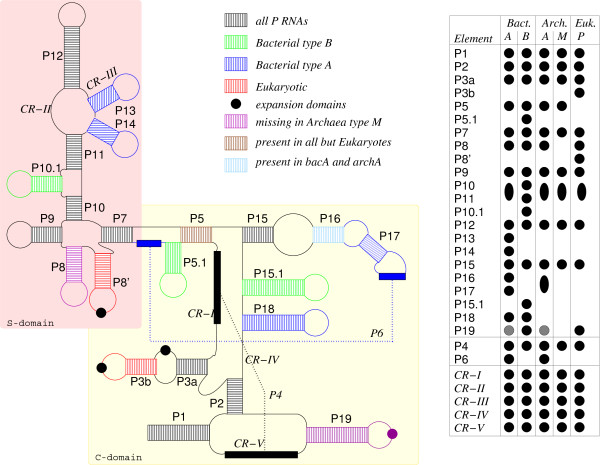
**Schematic drawing of the consensus structures of RNase P RNA (adapted from **[34,46-48]**)**. The table indicates the distribution of structural elements. Fungi type A conforms to the major eukaryotic distribution, while fungi type B structures have an additional P8' stem. A black circle in the table represents the occurrence of a particular element. Grey circles indicate features with extreme variations in some species. An ellipse indicates that two elements are merged and cannot be separated unambiguously.

The length of most RNase P RNA lies between 250 nt and 550 nt. It is divided into two structural domains: The S-domain repsonsible for binding and the catalytically active C-domain [27], see Figure 1. The secondary structure of RNase P RNA consists of up to 19 conserved stems, denoted P1 to P19, of which P7 to P14 form the S-domain, which is flanked by the C-domain [28]. There are five regions with strong sequence conservation, designated CR-I to CR-V, including the P4 pseudoknot composed by CR-I and CR-V [29].

The RNase P RNA structures can be broadly assigned to five subfamilies: the bacterial types A and B (bacA and bacB), the archaeal types A and M (arcA and arcM), and a single eukaryote group (nucA) [30]. In addition, two eukaryotic subtypes in fungi (fugA & fugB) can be identified [31]. The types arcA and bacA, which have been identified as ancestral states [32], cover the majority of microbial *rnpB *genes, forming diverse sets in terms of both sequence and structure variation. The derived types arcM and bacB, in contrast, have more uniform members. The diversity is largest among eukaryotic RNase P RNA genes. In eukaryotes, RNase P RNA is transcribed by polymerase III [33]. The human promoter elements were described recently [34] to contain TATA-box, PSE, Oct and SP1/SPH elements within 100 nt upstream of the transcription initiation site. A comparison of all eukaryotic promoter elements showed weak similarities only in the TATA box.

In this contribution we are concerned with the detection of RNase P RNA genes in genomic data from all domains of life. In [29], a pattern matching based pipeline for efficient *rnpB *gene prediction has been proposed. It is not applicable to large-scale database searches in practice, however. Here, we present Bcheck, a wrapper, to perform efficient *rnpB *gene prediction by combining the fast filtering with rnabob[17] and the sensitive validation by Infernal. The construction of such a method entails two tasks: the design of an efficient yet sensitive descriptor model (DM) that acts as a filter, and the derivation of a sensitive statistics covariance model (CM). Both components are based on a careful analysis of published RNase P RNA sequences and structures. The success of Bcheck depends on the efficiency and predictive power of both models, as well as a sensible wrapping algorithm that optimizes the interplay of DM and CM.

## 2 Results

### 2.1 Algorithm and models

The construction of effective models of RNase P RNA genes is a non-trivial task because of the lack of strong family-specific conservation. Our strategy was to first classify the training sequences into the seven sub-families identified in the literature: arcA, arcM, bacA, bacB, nucA, fugA and fugB. The training set consists of sequences from the RNase P RNA Database [35] with intact secondary structures annotated, and additional RNase P RNA sequences from the Rfam and from two recent publications [23,36]. A set of randomized decoys as well as randomized genomic sequences were constructed using ushuffle[37] in order to determine the false positive rates.

The training of both DM and CM requires structural alignments, whose quality is crucial for both the automatic learning procedures of the Infernal CMs and the manual construction of the DMs. We adopted a multi-step strategy: The RNase P RNA sequences were first divided into structural elements, then base-pairing regions were structure-aligned manually, and loop regions were sequence-aligned by means of MUSCLE[38]. Local alignments were then recombined into a "raw" global alignment for each subfamily. These alignments contained errors, mainly caused by local foldings which were not fitting to the conservation patterns shared by the majority of members. We applied RNAfold [39,40] to check the thermodynamic plausibility of local structure elements. Construction of DMs started with these alignments. In the course of DM construction, outliers were temporarily removed from the alignments, searched with the preliminary DMs, which provided additional information to guide the re-insertion of the outlier into the alignment.

Since efficiency was the major focus of DM training, we focused on selected features of local regions. To gain consensus sequence information, each alignment column was summarized and assigned with standard, "ambiguous" IUPAC nucleotides (taking into account every nucleotide appearing in the column) or the gap character (whenever the column contained at least one gap). The sequence was edited to take established structural knowledge into account. The resulting consensus sequence was then annotated in the alignment.

**Figure 2:**
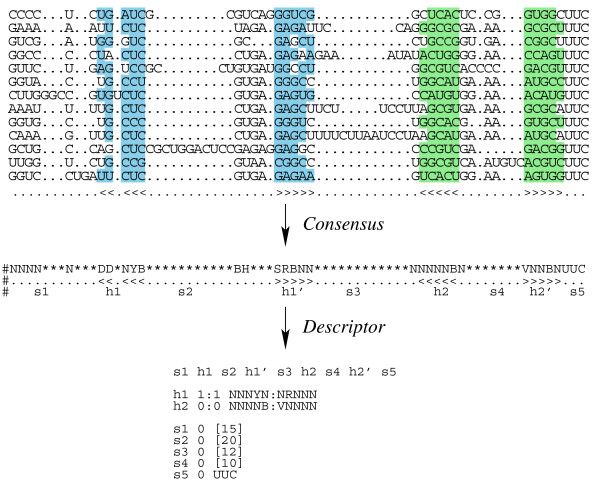
**Construction of a descriptor model (DM)**. A simple example based on a partial RNase P RNA gene sequence is shown here. The refined alignment columns are annotated with consensus structural and sequence information. The DM is then constructed by manual inspection of the best-conserved regions, taking into account both sequence and structure variation observed in the alignment.

The RALEE mode [41] in the emacs editor was used for visually inspecting alignments, consensus structures and conservation patterns. Regions with rich conservation in sequence and/or structure were selected for inclusion in the descriptor. A simple example of constructing DM from alignment is shown in Figure 2. The DM for RNase P RNA genes mainly consists of the S-domain and its flanking conserved sequences. Once the feature selection was completed, we carried out a interactive process between DM building and DM testing to adjust the parameters of feature variables balancing between false positive rate and efficiency. Among several descriptor languages we chose rnabob as search engine for our DMs because of its convenient syntax and its speed.

For the subfamilies arcA, bacA, and nuc with strong variation, we constructed two variants, "DM selective" and "DM general", with different parameter settings. The selective DMs miss a few aberrant RNase P RNA genes, while the "DM general" models have a larger false positive rate. For each subfamily, only one CM is needed and automatically generated based on global structural alignment using the tools of the Infernal package.

**Figure 3:**
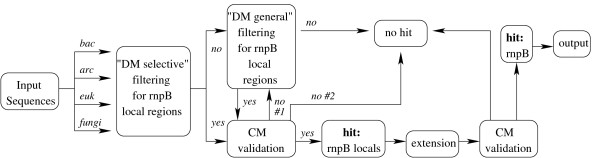
**Bcheck**** wrapping algorithm follows *local to global *and *selective to general *strategy**. See text for details. arc - archaea descriptor, bac - bacterial descriptor, fungi - fungal descriptor, euk - eukaryotic descriptor, DM - Descriptor Model, CM - Covariance Model.

The Bcheck wrapping algorithm takes the strategy of *local to global and selective to general*, Figure 3. At first, subfamily-specific DMs locate candidate genes. If no valid hit was produced by the "selective" model, the corresponding "DM general" is applied. Then the CM is applied in local alignment mode to validate the candidate. Valid hits, i.e., those recognized by the CM, are extended by 150 nt and 300 nt at 5' and 3' ends, respectively, and fed to the CM in global alignment to produce better estimates of the ends. At both phases, an *E*-value threshold of *E *≤ 10^-10 ^must be reached.

To distinguish functional copy and pseudogene of eukaryotes, we analyzed their promoter regions. For this purpose we aligned 100 nt upstream of Polymerase III transcripts of the same organism and compared the RNase P RNA gene predictions. While the absence of a recognizable promoter signal does not prove that the RNase P RNA gene is not functional, we have adopted a conservative policy and include only bcheck results for which the presence of a promoter signal provides additional confirmation.

### 2.2 Applications

#### 2.2.1 Prokaryote rnpB genes in GenBank

We used Bcheck to scan the genomic sequences of 956 bacteria and 68 archaea organisms from GenBank. The entire computation, which surveyed 3.1G of input sequence, took approximately 4 hours to complete with a single core of 2.4 GHz Intel(R) Core(TM)2 CPU. Bcheck produced one hit per organism for 98% (1005) of organisms, see Table 1. The default algorithm yielded no prediction in 29 organisms, for 10 of which a direct CM search was successful. These 10 RNase P RNA genes are distributed over all 4 prokaryotic sub-families. The main reason of failure is because of unusual local variations, e.g. bulges or point mutations, which cannot be captured by the present DMs. Bcheck predictions for three members of the phylum *Chloroflex *(*Roseiflexus castenholzii, Roseiflexus RS-1*, and *Chloroflexus aggregans*) are only partial *rnpB *regions including partial-P11, P12 and junctions between two stems. After removing duplicate sequences from closely related strains, we obtained 777 unique *rnpB *genes of which 45 belong to arcA, 10 to arcM, 621 to bacA, and 101 to bacB, see Table 3 below.

**Table 1:** Summary of predicted microbial *rnpB *for GenBank genome data set.

Domain	known	unknown	total	CM only
Eubacteria	365/365	581/591	946/956	7/946
Archaea	22/22	37/46	59/68	3/59

Total	387/387	618/637	1005/1024	--

"Known" refers to organisms with annotated *rnpB *genes, whereas "unknown" refers to organisms with no annotated *rnpB *genes.

The GenBank files contained annotated *rnpB *genes for 365 bacteria and 22 archaea, all of which were among the Bcheck predictions. We then compared start-end positions of Bcheck predictions and GenBank annotations. Only 25% of the annotations agree within a discrepancy of 5 nt or less at both ends.

Inspection of sequences and predicted secondary structures shows that the published sequences are in general incomplete, lacking nucleotides at the 5' end of P4 and 3' end of P4: At the 5' end, 66% known annotations miss flanking regions of P4, ranging from 30 to 90 nucleotides. At the 3' end, 56% known annotations miss flanking regions of P4', ranging from 10 to 20 nucleotides. A few of the GenBank annotations, furthermore, have promoter or terminator sequences included. Bcheck thus provides a substantial improvement also of the existing annotations in most cases.

The published annotation is more accurate than the Bcheck prediction only in a single case: *Roseiflexus RS 1*. In five cases, the published annotations and the Bcheck results differ dramatically. In order to evaluate these cases further, we employed the CM model of the Rfam, which supported the authenticity of the Bcheck predictions, Table 2.

**Table 2:** Evaluation of the five major discrepancies between GenBank annotation and Bcheck results.

Organism	**Rfam**** CM scores**			Discrepancy
	**GenBank**	**Bcheck**		
*M. acetivorans*	-63.35	167.88		P1, P7
*A. cellulolyticus*	-132.16	221.11		all
*E. coli(CFT073)*	-72.51	282.72		most
*R. typhi wilmington*	-97.66	264.53		P1, P3, P9, P10
*B. halodurans*	-110.59	300.87		P1, P9

Rfam scores are bit-scores for Infernal using Rfam's CM models. The discrepancies column lists features missing in the region annotated in GenBank.

**Table 3:** The subfamily distribution of microbial *rnpB*. No hit was obtained with any of the eukaryotic DMs.

Data set	arcM	arcA	bacB	bacA	total
GenBank	10	45	101	621	777
GOS	0	49	3	2857	2909

#### 2.2.2 RNase P RNA genes in metagenomic sequences

The GOS metagenomic sequences were obtained from the CAMERA project [42]. Due to the taxonomic uncertainty of the GOS data set, all models of archaea, bacteria and eukaryotes were applied to search over 10 million sequences comprising about 18 G. No hit was produced by any of the eukaryotic models.

In total, Bcheck predicted 4675 *rnpB *genes with median *E*-values of 10^-78^. In 211 cases two models overlap. In these cases there is a clear difference in *E*-values, so that the assignment to domains is unambiguous in all positive cases. After duplication removal, 2909 *rnpB *sequences are unique, 2857 of which belong to bacA, 49 to arcA, 3 to bacB, but none for arcM, see Table 3.

**Figure 4:**
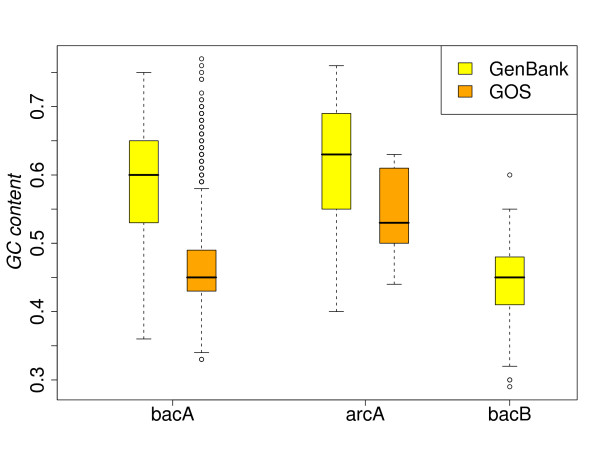
**Comparison of GC contents in the GOS and ****GenBank**** data sets**. The statistics were calculated based on unique genes with intact secondary structures. The difference of GC-content is particularly obvious in the bacteria domain.

The ancestral types arcA and bacA are clearly predominant in both GenBank and GOS data set. In the marine samples, the number of bacA *rnpB *genes exceeds 95%. We compared *rnpB *sequences from two datasets with respect to their GC content, Figure 4. Differences are particularly obvious in bacteria, where the majority of GOS bacA sequences have low GC-content, while the median GC content of GenBank* rnpB *is high, with ≈ 0.6. The GC content of RNaseP RNA genes seems to be influenced by the environment, which agrees to the findings of Foerstner et al. [43], showing the GC content of complex microbial communities seems to be globally and actively influenced by the environment.

**Figure 5:**
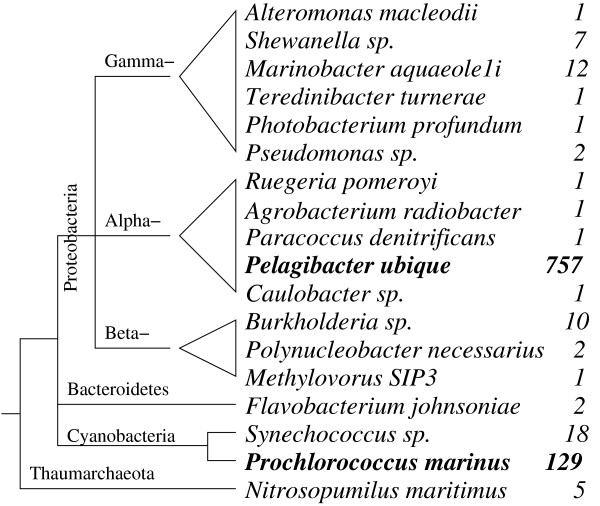
**Phylogenetic distribution of *rnpB *genes detected in the GOS data set**. 99.5% of the sequences are of bacterial origin, with three quarters deriving from *Pelagibacter ubique *and another 13% coming from *Prochlorococcus marinus*. Only 5 hits are of Archaeal origins.

Obtaining a view of the taxonomic distribution within a microbial community is one central objective of metagenomic analysis. We used the detected *rnpB *genes as a marker to infer the taxonomic distribution of GOS samples. We used blast to find the closest orthologs of 2909 unique GOS *rnpB *genes among the 777 GenBank sequences using an *E*-value threshold of *E *< 10^-50^. High scoring orthologs are found for 1003 GOS *rnpB *genes. 914 of which have only one ortholog per gene making species assignments possible, while 39 have multiple orthologs, all coming from the same genus, making genus assignments possbile. These species assignments and genus assignments are shown in Figure 5. The identified organisms are mostly bacteria belonging to the three phyla proteobacteria, cyanobacteria and bacteroidetes. Only a single archaeon, *Nitrosopumilus maritimus*, is recognized. Among bacteria, most sequences belong to *Pelagibacter ubique *(75%) and *Prochlorococcus marinus *(13%). For 1906 GOS *rnpB*s (66%), no close homologs are known, suggesting that they derive from unknown species. Of these, 1859 (97.5%) belong to the bacA subfamily, 44 (2.3%) to the arcA subfamily, and 3 (0.1%) to the bacB subfamily.

#### 2.2.3 RNase P RNA genes in eukaryotic genomes

We investigated 237 eukaryotic genomes, Table 4. Of the previously annotated genes, we recovered 84 of 85 metazoan and 19 of 21 fungal RNase P RNA genes. We miss the *Otolemur garnetti *sequence because of a 3 nt insertion within the highly conserved P4, which is used as a block in most descriptors. For the two related fungi *Coprinus cinereus *and *Laccaria bicolor *hypothetical RNase P RNA genes have been reported [23]. However, the E-values assigned by the Bcheck CM is mediocre, and the insertions of several 'U's within the 'GAA' motif of the P4 5' region as well as point mutations cause failure of the Bcheck DM. On the other hand, compared to [23,44]Bcheck made novel predictions for 32 fungi and 4 metazoans (*Meloidogyne hapla, Aedes aegypti, Canis familiaris*, and *Taeniopygia guttata*), as well as the choanoflagellate *Monosiga brevicollis*.

**Table 4:** Summary of predicted RNase P RNA genes in eukaryotes. "Known" refers to organisms with annotated RNase P RNA genes, whereas "unknown" refers to organisms with no annotated RNase P RNA genes.

	known	unknown	Sum
Metazoans	84/85	4*/13	88/98
Fungi	19/21	32/49	51/70
Heterokonts	0/0	0/6	0/6
Plants	0/0	0/30	0/30
Other Eukaryotes	7/12	1/21	8/33

Sum	110/118	37/119	147/237

* No common promoter signals observed: 3 out of 4.

Strong promoter signals were identified for *Meloidogyne hapla *and *Monosiga brevicollis*, supporting that these candidates are functional copies. For 36 metazoan genomes, Bcheck made multiple predictions. In at least 16 cases, additional predictions are highly similar to the functional copy. In genome sequence assembly, the reads originating from different copies of a repeat appear identical and cause assembly errors.

Therefore these predictions seem to be due to assembly errors rather than constituting true paralogs. In the other 20 cases differences in the flanking regions and the RNase P RNA gene itself indicate that we see the results of gene duplications. Even though the promoter might be specific for an organism, it may differ from other polymerase III transcripts within one species. In each of these cases, a presumably functional RNase P RNA like promoter structure was found for only one of the copies. Similar duplication patterns are observed in closely related primate, fish and rodent. For instance, both *Homo sapiens *and *Pan troglodytes *have functional copies on chromosome 14 and a pseudogene on chromosome 4. Among teleosts, both *Danio rerio *and *Gasterosteus aculeatus *have their functional copies and pseudogenes on the chromosome 2. In rodent family, *Rattus norvegicus *and *Mus musculus *have the pseudogenes spreading on at least 4 different chromosomes.

Novel RNase P RNA genes were detected by Bcheck in many fungi, which had not been analyzed in much detail so far. Only eight sequences which were reported before were not recognized by Bcheck. In 119 sequences Bcheck found 37 novel RNase P RNA genes. The remaining 82 genomes are either unfinished drafts, so that the RNase P RNA is not contained in the data, or they belong to clades where RNase P RNA may be absent. In plants, red algae, and heterokonts RNase MRP RNA, an ancient paralog of RNase P RNA, is well described [23,34]. One may speculate that it substitutes for RNase P RNA in these clades, in particular given that multiple copies of RNase MRP RNA are present in plant genomes. A recent, detailed overview of the evolution of RNase P RNA and its associated proteins can be found in [23,44]. An incomplete genome assembly explains e.g. the deviant RNase P RNA in the genome of the elephant (*Loxodonta africanus*), which shows a canonical sequence interrupted by a run of Ns in the latest assembly (Loxafr3.0). On the other hand, we suspect that we missed the RNase P RNA in some fungi and in some of the basal eukaryotes because of highly divergent sequence and secondary structure.

### 2.3 Software, webserver, and database

Bcheck was written in Python (version 2.5.2). Input consists of DNA or RNA sequences in fasta format, predictions are output with fasta format or with secondary structure annotated. Besides the default search algorithm, Bcheck also gives the option for searching with CM only. However, CM-only search is at least 100 times slower.

We set up a Bcheck webserver to facilitate online RNase P RNA gene prediction. A searchable rnpB database was developed, including genes for 1005 microbial organisms, 147 eukaryote organisms and 4756 GOS sample sequences. The predicted pseudogenes for eukaryote organisms are also included. The "rnpB database" uses a hierarchical tree structure, consisting of 5 tables, implementing preorder tree traversal algorithm to process the query efficiently. blast is also offered in the server for homology search against the database compromising 777 unique *rnpB *genes. The server can be accessed at http://rna.tbi.univie.ac.at/bcheck. The Bcheck-pipeline can also be downloaded from the same location for local use in a Linux environment.

## 3 Discussion and conclusions

The rapidly increasing size of sequence databases requires efficient tools for data analysis. In particular, homology annotation of small ncRNAs, with their short and often poorly conserved sequences poses a severe problem for large-scale annotation. Here, we describe Bcheck, an efficient pipeline to determine RNase P RNA genes across all three domains of life. In order to deal with the high variability of the RNase P RNA sequences and structures, we employ descriptor-based models specific for sub-families instead of a single pattern to construct more efficient filters. In the second step, improved covariance models are used to validate the candidates from the DM step and to determine nearly exact gene boundaries.

With Bcheck, we were able to determine the RNase P RNA genes in 59 out of 68 archaea, 946 out of 956 bacteria, and 147 out of 237 eukaryotes. 61% of the prokaryotic sequences and 25% of eukaryotic results were not annotated previously. The quality of the predicted *rnpB *genes is much better than a large fraction of the - usually blast-based - annotation available through GenBank. The size and diversity of eukaryote genomes brings with it a particular challenge in finding RNase P RNA genes, because this diversity is reflected in many aberrant features of the RNase P RNA itself. Using the fungi-specific DMs, we uncovered 32 previously unannotated sequences, which are downloadable from the Bcheck server. As in previous studies, we did not find RNase P RNA candidates in plants and Heterokonta.

Since Bcheck is more than 100 times faster than the direct application of Infernal (version 1.0), it is suitable in particular as a tool to screen large high-throughput sequencing data. With only a handful of false negatives (10 out of 1005 prokaryotes), Bcheck provides a highly efficient way to annotate newly sequenced genomes. A particular strength of Bcheck is its applicability to metagenomics data.

**Figure 6:**
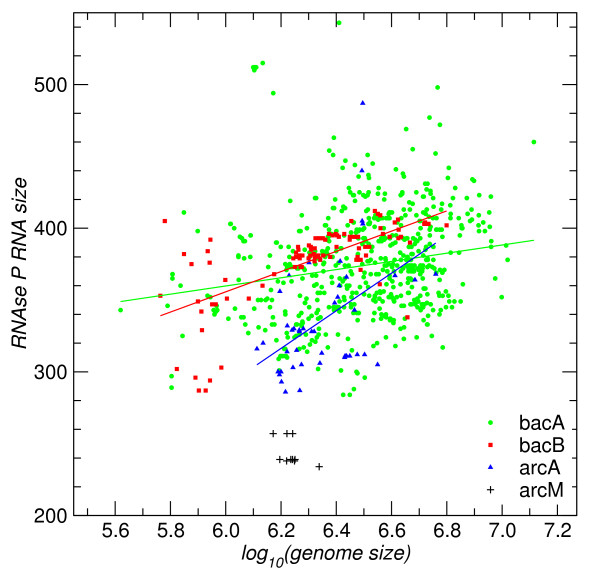
**Correlation of the size of RNase P RNA genes with genome size**. For bacA (*ρ *= 0.16), bacB (*ρ *= 0.66), and arcA (*ρ *= 0.47) there is a weak but significant positive correlation. The few sequences of type arcM are significantly shorter and are restricted to genomes in a very narrow size range.

Among the 19 prokaryotic genomes for which Bcheck failed to detect a candidate, 15 have a size below 2.0 Mbp. One of them, *Nanoarchaeum equitans*, is among three organisms having extremely condensed genomes with length even below 0.5 Mbp. *Nanoarchaeum equitans *appears to have lost its RNase P RNA through genomic rearrangement at tRNA genes under the pressure of genome condensation [18]. In Figure 6 we summarize the correlations between genome size and the size of prokaryotic RNase P RNA. Even though there is no strong correlation indicated in arcA and bacA subfamilies, the evolutionarily younger bacB and arcM seem to be more strongly affected by changes in genome size.

At present, Bcheck models were built on the conserved sequence and secondary structure features of a large sample of RNase P RNA genes. Conceivably, the predictive power of the pipeline could be improved further by including additional information. For instance, promoter and terminator regions might be utilized. A recent survey for 7SK RNAs capitalized largely on the conserved features of the characteristic pol-III promoter signals of this ncRNA class [45]. A similar strategy might allow a further relaxation of the DM pattern in favor of a second filter utilizing the promoter and terminator motifs.

## Authors' contributions

ILH designed the study. DY constructed descriptors, designed and implemented the pipeline. MM prepared the training sets for eukaryote models and performed eukaryote hit validation with promoter analysis. All authors collaborated on analyzing the data and writing the manuscript. All authors have read and approved the final manuscript.
